# Plasma neutrophil gelatinase-associated lipocalin as a single test rule out biomarker for acute kidney injury: A cross-sectional study in patients admitted to the emergency department

**DOI:** 10.1371/journal.pone.0316897

**Published:** 2025-01-10

**Authors:** Vicky Jenny Rebecka Wetterstrand, Martin Schultz, Thomas Kallemose, André Torre, Jesper Juul Larsen, Lennart Friis-Hansen, Lisbet Brandi

**Affiliations:** 1 Department of Clinical Biochemistry, North Zealand University Hospital, Denmark; 2 Department of Geriatrics, Herlev University Hospital, Herlev, Denmark; 3 Institute of Clinical Medicine, University of Copenhagen, Copenhagen, Denmark; 4 Department of Clinical Research, Copenhagen University Hospital Amager and Hvidovre, Hvidovre, Denmark; 5 Department of Emergency, North Zealand University Hospital, Denmark; 6 Dept of Clinical Microbiology, Rigshospitalet, Copenhagen University Hospital, Copenhagen, Denmark; 7 Department of Endocrinology and Nephrology, North Zealand University Hospital, Denmark; Faculdade de Medicina de São José do Rio Preto, BRAZIL

## Abstract

**Objectives:**

Acute kidney injury (AKI) is a syndrome with high mortality and morbidity in part due to delayed recognition based on changes in creatinine. A marker for AKI based on a single measurement is needed and therefore the performance of a single measurement of plasma neutrophil gelatinase-associated lipocalin (pNGAL) to predict AKI in patients admitted to the emergency department was tested.

**Methods:**

Samples from the Triage study which included 6005 consecutive adult patients admitted to the emergency department were tested for pNGAL. The optimal cutoff for pNGAL was determined by the AUC and compared to AKI based on creatinine using different estimations of the premorbid kidney function.

**Results:**

In 4833 patients, two or more plasma creatinine (pCr) measurements were available allowing the detection of AKI. The highest prevalence of AKI (10%) was found when defining AKI as an increase in pCr ≥26.5 μmol/L from the prior year’s mean pCr. At these conditions the AUC for pNGAL to predict AKI was 85% giving an optimal cutoff of 142.5 ng/mL with a negative predictive value of 0.96, a positive predictive value of 0.35, a specificity of 0.87 and a sensitivity of 0.70.

**Conclusion:**

The study illustrates that the value of a single measurement of pNGAL is primarily in excluding AKI whereas it`s poorer in predicting the presence of AKI. When diagnosing AKI with pCr the optimal baseline pCr level is the mean of available pCr (mb-pCr) measurements from up to a year prior to the current event.

## Background

Acute kidney injury (AKI) is a common syndrome with a globally increasing prevalence and is associated with a high mortality and morbidity [1–3] which is in part due to inadequate and/or delayed recognition [1]. To standardize diagnosis and improve outcomes a uniform definition of AKI and kidney function baseline was published in the “Kidney Disease: Improving Global Outcomes” (KDIGO) [4]. However, in daily clinical practice there is still some variation in the interpretation which challenges a homogeneous clinical pathway and early intervention.

In a population study, 21% of the patients admitted to the hospital developed AKI during their stay and found that the more severe the AKI was, the greater risk of death and in-hospital mortality [5]. Moreover, patients who developed AKI had longer in-hospital length-of-stay and increased hospital readmissions. Furthermore, patients with a prior AKI episode had almost 30% greater risk of being readmitted with AKI within a median of 0.6 years, compared to the first episode [6]. In addition, patients who developed AKI subsequently had an increased risk for developing chronic kidney disease (CKD) [3, 7]. The value of early identification of AKI and ensuring intervention was demonstrated in a study in which the electronic laboratory result system alerted the staff of acute changes in creatinine (Cr) and subsequent risk of AKI [8]. This resulted in faster and better management of AKI, reduced the length of stay in hospital and improved mortality rates. Overall, this highlighted the need for and value of improved and simplified recognition of AKI.

Due to the inherent delays and limitations of functional biomarkers such as pCr [9] alternative injury-biomarkers that responds more rapid than functional biomarkers [10] have been proposed. The iron binding 21–25 kD lipocalin superfamily protein neutrophil gelatinase-associated lipocalin (NGAL) is one kidney injury biomarker. After an AKI event NGAL is expressed in the tubular epithelium of the loop of Henle and collecting ducts in the kidneys. In the kidney, the NGAL expression increases in response to noxious stimuli, for instance ischemia–reperfusion damage and conditions predisposing to AKI [11]. Increased levels of plasma NGAL (pNGAL) are detectable within six hours from an AKI event [12] and the concentration of both pNGAL and urine NGAL (uNGAL) appears to correlate with the degree of tubulointerstitial injury, indicating the degree of kidney function [13, 14]. NGAL represent the renal cells response to an intrinsic AKI event. In AKI, the increase in plasma NGAL levels is mainly caused by a rapid induction of NGAL expression and NGAL release from the kidneys due to acute tubular damage, systemic inflammation, and decreased reabsorption capacity, resulting in impaired clearance and accumulation in plasma and urine. In the acute phase of AKI, the reduced clearance of NGAL plays a minor role. In contrast, in CKD, prolonged filtration impairment leads to a progressive buildup of NGAL in the plasma (due to reduced filtration and excretion), with levels correlating to disease severity and renal function decline. In CKD there is no/less increase in renal NGAL synthesis. Thus, pNGAL clearance is closely linked to kidney function status and serves as a sensitive marker of renal injury in both AKI and CKD contexts [15].

Further, pCr is also affected by pre- and post-renal AKI events and in NGAL therefor also reflects extra-renal diseases that also leads to a decreased glomerular filtration rate (GFR) [16]. NGAL is secreted either as a monomer (mainly from renal tubular epithelial cells) or as a dimer (mainly form neutrophil granulocytes) [17]. Varying the assays specificity, for either the monomer or the dimer, making the assays predominantly sensitive to AKI (the monomer) or infections (the dimer) [18].

Most studies done on NGAL, as a kidney injury biomarker, have been in settings where patients have suffered from a specific disease or have been selected for a specific clinical condition [19–21]. In contrast, in the emergency department (ED) patients are unselected and suffer from both acute and chronic diseases. We therefore wanted to examine the performance of a single NGAL test for diagnosing AKI at admission to the ED compared to the standard AKI diagnosis based on pCr.

## Methods

### Study design, setting and participants

The study was a retrospective cross-sectional study based on samples collected as part of the Triage study biobank that included patients admitted to the ED, at North Zealand University Hospital, from September 5^th^, 2013, to December 6^th^, 2013 [22]. The original study [22] included 6005 consecutive patient admissions from which both blood sample and triage information was collected, why the authors for the current study had no access to information that could identify individual participants during or after data collection; all patients with consent and an age of ≥ 18 years, admitted to the ED during inclusion period, were included. Obstetric patients were excluded due to an immediate admission to the obstetric department. The blood samples were subsequently stored at a biobank [22]. North Zealand University Hospital is part of the Capital Region of Denmark and conducts medical-, surgical-, level-2 trauma-, emergency interventions and intensive care, around the clock [23].

### Data collection and NGAL testing

The blood samples, originally collected for the TRIAGE study [22] and stored as a biobank, were taken within the first 60 minutes after admission. pCr values up to one year before admission was retrieved from the patients’ laboratory results records through the TRIAGE study data.

The Triage study biobank samples were stored at -80°C and NGAL was measured from August 4^th^, 2021, to January 3^rd^, 2022 using The NGAL ST001 assay (BioPorto, Hellerup, Denmark) (measuring range 50 ng/mL to 3000 ng/mL, CV% = 14%) [24] on the Cobas 8000 platforms c701 module at the Department of Clinical Biochemistry at Bispebjerg hospital. The ED routine pCr was measured using the Dimension Vista^®^ 1500 enzymatic creatinine assay (Siemens Diagnostics, Terrytown, NJ) at North Zealand University Hospital [22].

### Definition criteria and staging of AKI

**Fig 1:**
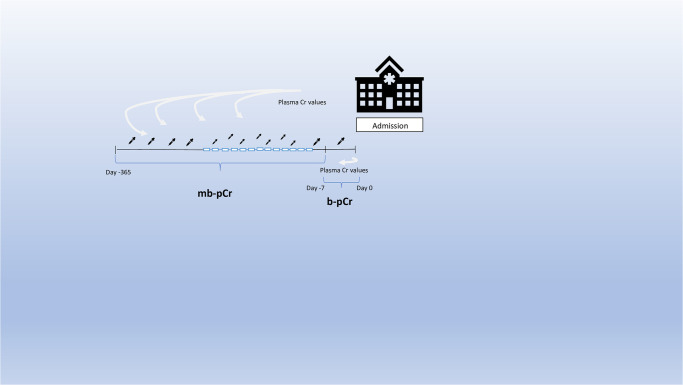
A timeline illustrating the assessment of premorbid kidney function. A timeline illustrating the assessment of premorbid kidney function using baseline plasma creatinine (b-pCr) and mean baseline plasma creatinine (mb-pCr). If available, the lowest pCr measurement within seven days prior to study inclusion established the baseline. When no pCr measurement was available within this seven-day window, the mean of all pCr measurements taken up to 365 days before ED admission (mb-pCr) represented the kidney baseline.

Based on the creatinine measurement performed at admission to the ED the patients were divided into those who had AKI at admission or those who did not have AKI according to the KDIGO guidelines [4]. The primary outcome was the performance of diagnosing AKI based on a single pNGAL measurement. As demonstrated in Table 1a four different criteria definitions (as described in KDIGO-guidelines [4]) were used to diagnose AKI. If available the lowest pCr measured, within seven days prior to inclusion, was used as a measure for premorbid kidney function and used to define the baseline pCr (b-pCr) (diagnostic criteria (DC) II, Table 1a). However, the use of a single pCr measurement performed up to 365 days prior to an AKI event, in the absence of pCr measured within seven days, as a baseline for calculating the pCr delta value has been challenging (Fig 1). Instead, if a pCr had not been measured within the seven days prior to admission to the ED, the mean value of all pCr measurements (mb-pCr) performed up to 365 days prior to admission was used as premorbid kidney function [25, 26] (DC III and IV, Table 1a). Depending on the data available the same patient could be included in all four AKI-DCs groups. Only patients that could not be categorized into at least one of the AKI-DC were excluded. Urine output was not included in the study. mb-pCr and b-pCr were also used to investigate if there was a different AKI staging outcome, depending on how the premorbid kidney function was calculated (Table 1b and 1c).

**Table 1:** The different criteria used in the study for acute kidney injury (AKI) and their definitions.

**a.**	
**AKI diagnostic criteria (AKI-DC)**	**Definition**
I	≥ 26.5 μmol/L within 48 hours
II	≥ 1.5 fold baseline pCr (b-pCr)
III	≥26.5 μmol/L from mean baseline pCr (mb-pCr)
IV	≥1.5 fold from mean baseline pCr (mb-pCr)
**b.**	
**AKI staging using baseline pCr (b-pCr)**	**Definition**
1	≥ 26.5 μmol/L within 48 hours or ≥ 1.5–1.9 fold from b-pCr
2	≥ 2.0–2.9 -fold from b-pCr
3	≥ 3.0-fold from b-pCr
**c.**	
**AKI staging based mean baseline pCr (mb-pCr**)	**Definition**
1	≥ 26.5 μmol/L within 48 hours or ≥ 1.5–1.9 from mb-pCr
2	≥ 2.0–2.9 -fold from mb-pCr
3	≥ 3.0-fold from mb-pCr

Abbreviations: AKI-DC = acute kidney injury–diagnostic criteria; pCr = plasma creatinine;

b-pCr = baseline plasma creatinine; mb-pCr = mean baseline plasma creatinine

### Data from medical records

Data on gender, age, comorbidity, social status and hospital length of stay (LOS) was retrieved from the original TRIAGE study database [22] which had retrieved the data from patients`medical records.

### Ethics

The Danish ethical regulations were followed and approved by the Danish Data Protection agency (J. 2007-58-0015).

### Statistical method

Descriptive statistics were done by median and interquartile range (IQR) for numerical variables frequency and percentages for categorical variables. Chi-square and Wilcoxon sum-rank test was used to test for difference between the groups of patients examined. The p-value was adjusted for multiple comparisons using the Bonferoni correction.

Two statistical models, logistic regression and the cumulative logit model, were employed to assess NGAL’s ability to predict AKI. The logistic regression model differentiated between AKI and non-AKI, while the cumulative logit model was used to distinguish between the ordered AKI stages 1, 2 and 3.

Before fitting the cumulative logit models, the difference in mean NGAL between AKI stages 1,2 and 3 were compared visually by boxplots and tested by one-way ANOVA. Proportional odds assumption for the cumulative logit models were evaluated by Brant test.

After analyzing the two statistical models, we performed Receiver Operating Characteristic (ROC) analyses, to assess NGAL’s discriminatory value in relation to AKI for all four AKI-DC (I-IV) and for AKI staging. We used Youden index to determine the NGAL cutoff value. For AKI staging patients were categorized into two groups: AKI Stage 1 vs. AKI Stage 2 and 3, or AKI Stage 1 and 2 vs. AKI Stage 3. Area under the curve (AUC) was presented with 95% confidence interval (CI). An AUC with value 0.7–0.8 was considered as acceptable; 0.8–0.9 as excellent and > 0.9 as outstanding [27]. Sensitivity, specificity, and positive and negative predicted values from the ROC analyses were calculated.

All analysis were done using R 4.2.0(R Foundation for Statistical Computing, Vienna, Austria). Logistic regression was performed using the glm function from the stats package in R. Cumulative logit model was fitted using the vglm function from the VGAM package. The Brant Test was conducted using the polr function from the MASS package and the brant function from the brant package. ROC analysis was carried out using the roc function from the pROC package.

## Results

### Participants

**Fig 2:**
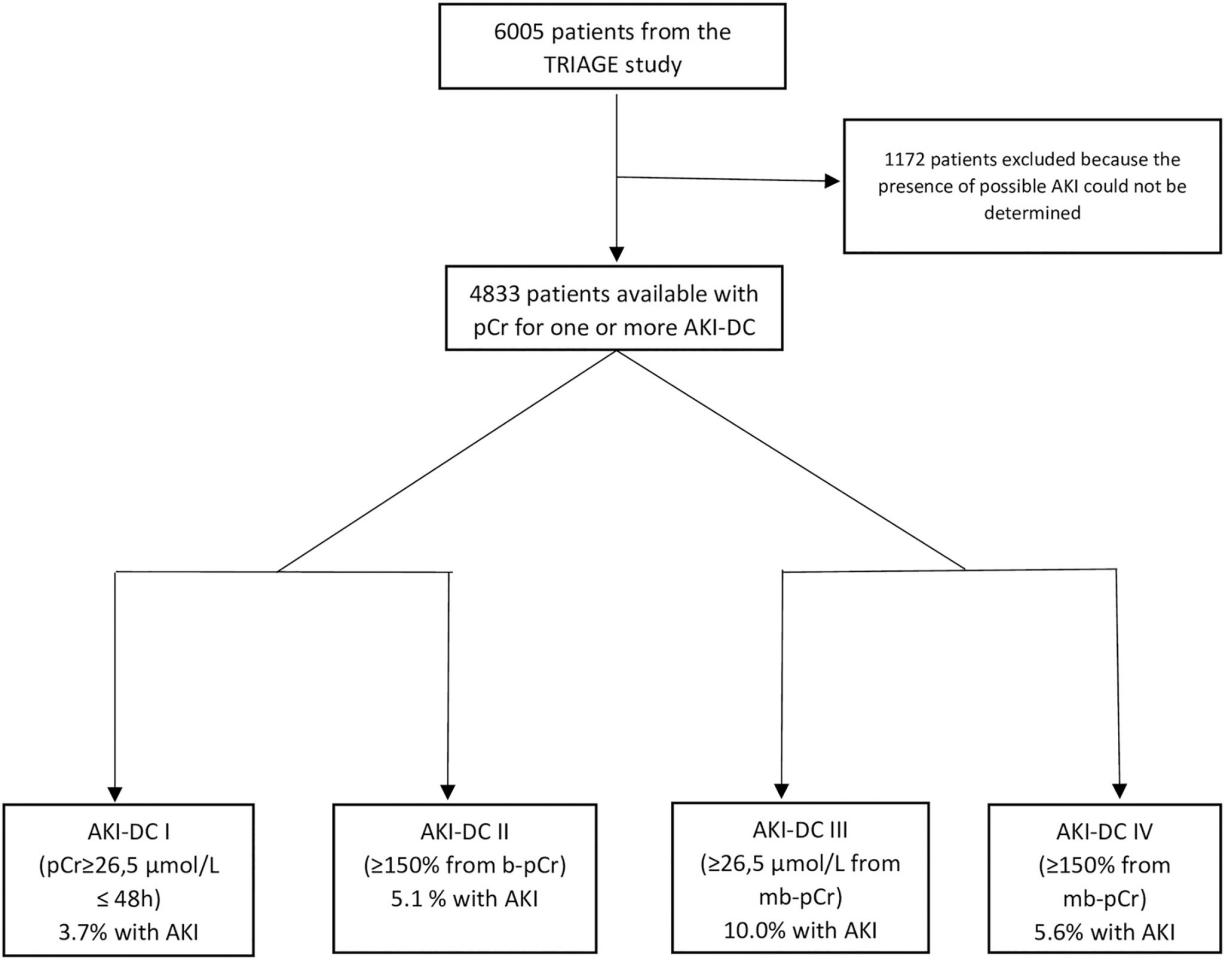
Flowchart over patient inclusion for the acute kidney injury (AKI) cohort and distribution between acute kidney injury- diagnostic criteria (AKI-DC) I- IV. Abbreviation: AKI = acute kidney injury; pCr = plasma creatinine; AKI-DC = acute kidney injury–diagnostic criteria.

**Fig 3:**
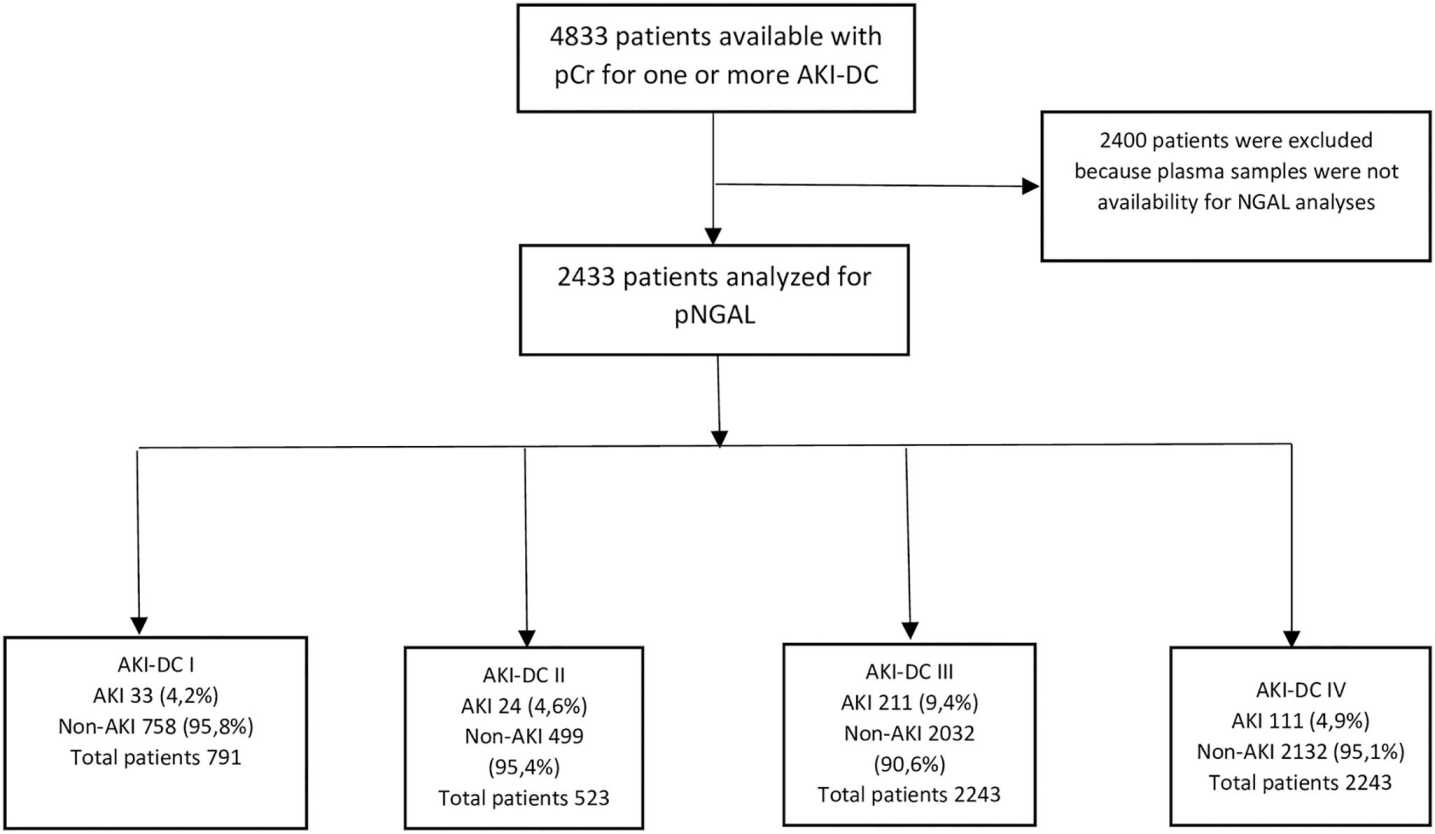
Flowchart over patient inclusion for the pNGAL cohort and distribution between acute kidney injury- diagnostic criteria (AKI-DC) I-IV. Abbreviation: AKI = acute kidney injury; non-AKI = non- acute kidney injury; pCr = plasma creatinine; AKI-DC = acute kidney injury–diagnostic criteria.

For 4833 patients out of the 6005 patients included in the Triage study it was possible to retrieve the additional pCr measurements from the patients record system needed to determine if a patient had AKI at admission according to at least one of the AKI-DCs (Table 1 and Fig 2)–from here on termed “the AKI cohort”. From the “AKI cohort” the AKI incidence, for each AKI-DCs (I-IV), was calculated (if possible) (Fig 2). In 2433 plasma samples (41% of the total number of included patients in the TRIAGE study [22]) from the AKI cohort pNGAL could be measured (the “NGAL cohort”) whereas in 2400 samples there was not enough plasma to perform the NGAL analysis and these patients were subsequently excluded from further analyses (Fig 3). To test if there was a difference in patients between the cohorts (Table 2), the prevalence of comorbidities was compared. A small but significant variation between groups (p<0.003) was found. The NGAL cohort had a higher prevalence in all comorbidities. Although statistically significant the variation between categories and AKI-DCs (S1 Table) are inconsequential in clinical practice.

**Table 2:** Demographics and comorbidities in the acute kidney injury (AKI) cohort and NGAL cohort.

	AKI cohort* (n = 3950)	NGAL cohort (n = 2433)	p-value
**Age in years (IQR)**	**60 (43: 75)**	**66 (50: 78)**	**<0.001**
**LOS, median days (IQR)**	**1 (0: 4)**	**1 (0: 5)**	**<0.001**
**Gender male, n (%)**	**1967 (49.8%)**	**1196 (49.2%)**	**0.638**
**Gender female, n (%)**	**1983 (50.2%)**	**1237 (50.8%)**	**0.638**
**IHD, n (%)**	**337 (8.5%)**	**285 (11.7%)**	**<0.001**
**CHF, n (%)**	**195 (4.9%)**	**179 (7.4%)**	**<0.001**
**Hypertension, n (%)**	**815 (20.6%)**	**604 (24.8%)**	**<0.001**
**Diabetes, n (%)**	**356 (9%)**	**326 (13.4%)**	**<0.001**
**COPD, n (%)**	**294 (7.4%)**	**238 (9.8%)**	**0.001**
**Kidney disease, n (%)**	**120 (3%)**	**113 (4.6%)**	**0.001**
**Liver disease, n (%)**	**75 (1.9%)**	**49 (2%)**	**0.818**
**Rheumatic disease, n (%)**	**72 (1.8%)**	**71 (2.9%)**	**0.005**
**Cancer, n (%)**	**463 (11.7%)**	**430 (17.7%)**	**<0.001**
**Living alone, n (%)**	**809 (20.5%)**	**542 (22.3%)**	**0.094**
**Domestic help, n (%)**	**353 (8.9%)**	**265 (10.9%)**	**0.012**
**Nursing home, n (%)**	**190 (4.8%)**	**174 (7.2%)**	**<0.001**

Abbreviation: LOS = length of stay; IHD = ischemic heart disease; CHF = cardiac heart failure;

COPD = chronic obstructive pulmonary disease

*Cohort on patients with AKI status and comorbidity data available

p-value significance set to 0.003 to adjust for multiple testing.

### The prevalence of AKI depends on the diagnostic criteria used

In the AKI cohort the highest AKI prevalence (nearly 10%) was found when using pCr increase ≥26.5 μmol/L from mb-pCr (AKI-DC III) while the prevalence based on the other three AKI-DCs varied between 3.7–5.6% (Table 3). Depending on the available pCr measurements AKI could be evaluated in 33% of the patients (791/2433) based on AKI-DC I, in 21% (523/2433) on AKI-DC II and in 92% (2243/2433) on AKI-DC III and AKI-DC IV (Fig 3). In patients where AKI-DC III and IV could be evaluated 31% and 33% had pCr available within 48 hours respectively 7 days from admission.

**Table 3:** Patient distribution of acute kidney injury (AKI) and non-AKI based on AKI- diagnostic criteria (AKI-DC) I-IV.

AKI-DC		Total(n)	AKI (n (%))
I	All	1657	61 (3.7%)
	NGAL	791	33 (4.2%)
II	All	1020	52 (5.1%)
	NGAL	523	24 (4.6%)
III	All	4445	446(10%)
	NGAL	2243	211 (9.4%)
IV	All	4445	249 (5.6%)
	NGAL	2243	111 (4.9%)

Abbreviation: AKI = acute kidney injury; AKI-DC = acute kidney injury–diagnostic criteria; non-AKI = non- acute kidney injury

### The cutoff for NGAL for detecting AKI defined by the four AKI-DCs

**Fig 4:**
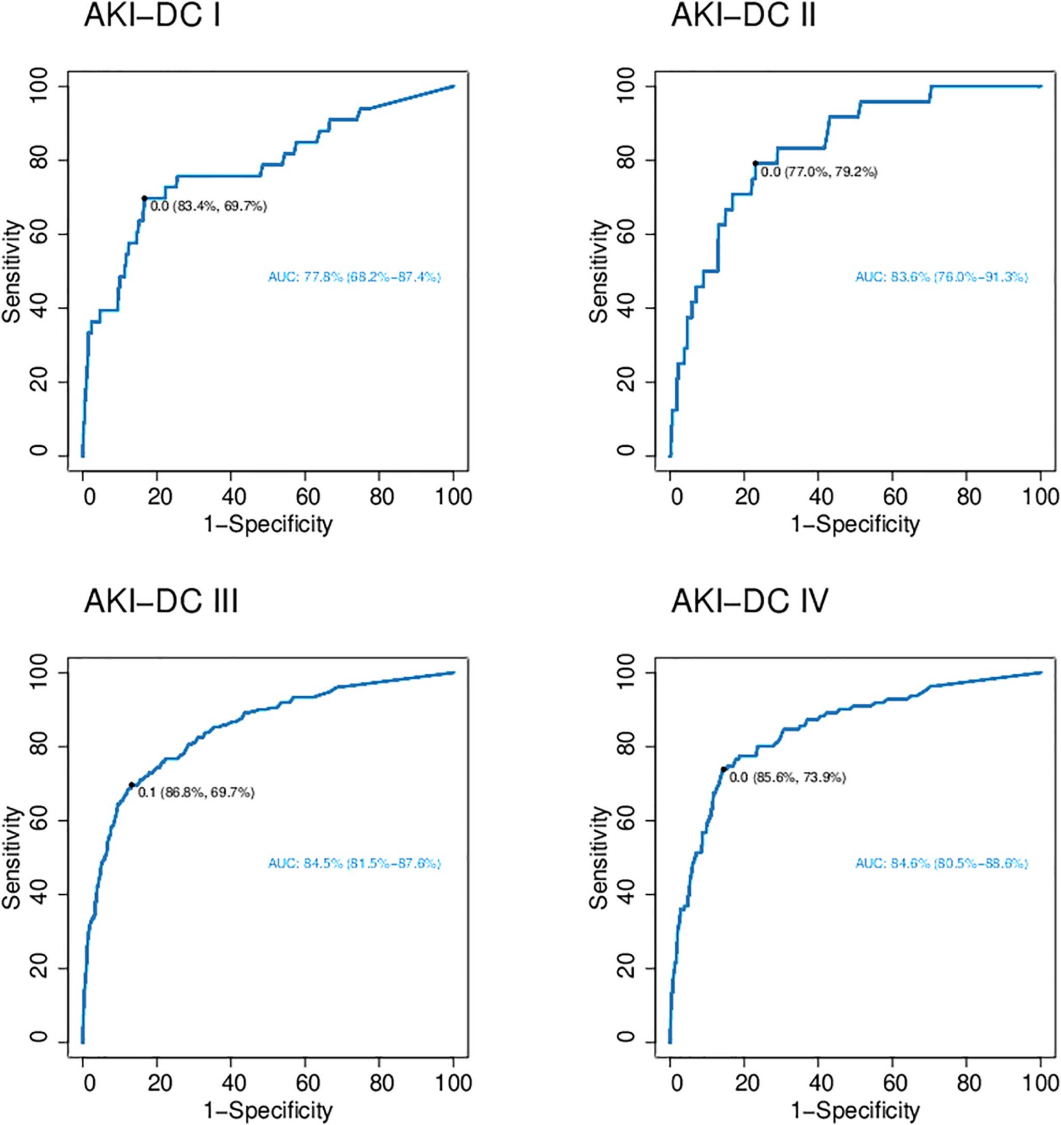
Receiver Operating Characteristic (ROC) curves for acute kidney injury- diagnostic criteria (AKI-DC) I-IV. ROC analyses were used to calculate area under the curve (AUC) for each AKI-DC (I-IV) to determine the optimal NGAL cutoff to diagnose acute kidney injury (AKI). AUC for AKI-DC I was acceptable and for AKI-DC II-IV excellent.

The AUC for prediction of AKI based on AKI-DC I and AKI-DC II using NGAL were acceptable to excellent (AUC of 0.78 and 0.84, respectively). For AKI-DC III and IV the AUC was 0.85 (Fig 4). The performance of pNGAL had higher specificity than sensitivity for AKI-DC I, III and IV. In all four AKI-DC pNGAL had high negative predictive value (NPV) (0.96–0.99) but a low positive predictive value (PPV) (0.15–0.35) which indicates that NGAL is an excellent biomarker for excluding AKI. The cutoff value for pNGAL varied between 132.5 to 192.5 ng/mL depending on AKI-DCs (Table 4); though when the AKI diagnosis was based on mb-Cr the cutoff only varies from 142.5 to 148.5 ng/mL.

**Table 4:** pNGAL performance at optimal cutoff values.

AKI-DC	Optimal threshold	NGAL Optimal Cutoff (ng/mL)	AUC (%)	Sensitivity	Specificity	PPV	NPV
I	0.035	192.5	78 [68, 87]	0.70	0.83	0.15	0.98
II	0.033	132.5	84 [76, 91]	0.79	0.77	0.14	0.99
III	0.085	142.5	85 [82, 88]	0.70	0.87	0.35	0.96
IV	0.042	148.5	85 [81, 89]	0.74	0.86	0.21	0.98

Abbreviation: AUC = area under the curve; PPV = positive predictive value; NPV = negative predictive value

Receiver Operating Characteristic (ROC) were conducted to analyze NGAL performance and calculate an NGAL cutoff value at the different AKI-DC (I-IV). The greatest AUC was found for AKI-DC III and IV. For all four AKI-DC NPV was greater than PPV and for AKI-DC I, III and IV specificity was higher than sensitivity.

### NGAL ability of AKI staging

In the patient who had AKI at admission and in which pNGAL could be measured it was possible to determine the KDIGO AKI stage based on b-pCr in 51 patients and for AKI staging based on mb-pCr in 213 patients (Table 5). Roughly 80% of the patients were stage 1, 15% stage 2 and 6% stage 3, both when using AKI staging based on b-pCr and when using mb-pCr.

**Table 5:** Patient distribution between acute kidney injury (AKI) stage 1,2 and 3.

Based on	Patients	AKI Stage 1 (n)	AKI Stage 2 (n)	AKI Stage 3 (n)	Non-AKI (n)	AKI (%)
b-pCr	All	80	18	3	2016	4.8%
	With NGAL value	40	8	3	975	5.0%
mb-pCr	All	362	65	29	3989	10.3%
	With NGAL value	170	29	14	2030	9.5%

Abbreviation: AKI = acute kidney injury; b-pCr = baseline plasma creatinine; mb-pCr = mean baseline plasma creatinine

There was a higher prevalence of AKI, at all stages, when AKI was diagnosed based on mb-Cr.

The boxplots of mean NGAL for the different AKI stages based on b-pCr seemed similar (S1 Fig) and the statistical differences was found insignificant by one-way ANOVA (p = 0.837). Therefore, no further statistical analysis was conducted for comparing AKI staging based on b-pCr. In contrast, for AKI staging based on mb-pCr the differences in NGAL distribution were seen (S1 Fig), which was also supported by significant one-way ANOVA (p <0.0001). However, these differences were most pronounced for stage 1 compared to 2 and 3. This was also reflected in the AUC in the cumulative ROC analysis done for mb-pCr. Though as demonstrated by the ROC curves pNGALs ability to discriminate AKI staging was on the lower end of acceptable (AUCs 0.70 and 0.71) (S2 Fig). Performance for AKI stage 1 vs. stage 2 and 3 show specificity and NPV above 0.8 with poor sensitivity and PPV. Stage 1 and 2 vs. 3 having sensitivity and NPV of 1 with poor specificity and PPV, suggesting the optimal cut-off, based on the Youden index, was obtain by the lowest value that ensures all stage 3 are found. This was also reflected in the vales for the cut-offs with a value of 445 ng/mL was 1 vs. stage 2 and 3 and 177 ng/mL for stage 1 and 2 vs. 3 (S2 Table).

## Discussion

This study found that evaluating kidney function and determining the presence of AKI at admission in a larger percentage of patients admitted to the ED is feasible when using changes in pCr from 365 to 7 days (mb-pCr) prior to admission, compared to a pCr value within the prior 7 days (b-pCr). The prevalence of AKI in the ED was found to be between 4–10% at admission, consistent with earlier studies performed in an ED setting [28–31].

Our findings indicate that determining AKI status based on a pCr value within 7 days prior to inclusion (b-pCr) is limited to a minor fraction of patients. Given the narrow time window required by AKI-DC I and II, AKI status was often indeterminable, highlighting the advantage of using mb-pCr for AKI diagnosis over a single measurement.

The prevalence of AKI was nearly 10% when diagnosed using an increase in pCr ≥26.5 μmol/L (AKI-DC III), but approximately 5% when using other diagnostic criteria (AKI-DC I, II, IV). This discrepancy raises questions about whether AKI incidence is underestimated with baseline pCr from the prior 48 hours or seven days or overestimated with mean pCr from the prior year. The results suggest that pCr may not be a reliable standalone biomarker for AKI, underscoring the potential utility of novel biomarkers like pNGAL. In the current study the pNGAL cutoff values for AKI ranged from 113 to 193 ng/mL. Similar values have been suggested in other studies [32–35] (S3 Table).

Further, the prevalence of AKI varies widely in the literature; presumably due to the differences in the AKI definitions used (KDIGO [4], RIFLE [36], AKIN [37]), the patient cohorts examined, and if the AKI diagnosis was exclusively retrieved from medical electronic records, as there is a large underreporting of AKI in clinical practice [38, 39], beyond the common pCr -affecting factors as age, body mass index and gender [4]. Depending on the setting, in which the patients are included, AKI prevalence has been found to range from 2.8% to almost 20% [29–31, 39–41].

In our observational cross-sectional ED study, a single measurement of pNGAL at admission demonstrated a low PPV but an excellent NPV, supporting its use as a rule-out biomarker for AKI at admission. To our knowledge, this has only been described in one previous study [12] whereas most studies have recognized and stated pNGAL as a useful biomarker for the prediction of AKI but not referred to a single measurement of pNGAL as an AKI rule out biomarker [28, 32, 33, 42].

A single pNGAL measurement demonstrated excellent discriminative ability for predicting AKI in three out of the four AKI-DC criteria (AKI-DC II, III, and IV). This approach is particularly an advantageous for potential AKI assessment, as it allows for diagnostic utility through exclusion. However, when using KDIGO criteria (AKI-DC I and II), this method could predict AKI in only up to 33% of the cases. Importantly, pNGAL minimizes the risk of misdiagnosis by excluding confounding factors that affect pCr, such as age, gender, BMI, and pre-/post-renal conditions. These benefits, combined with the early exclusion of AKI, makes pNGAL a valuable tool for patient admission in an ED setting.

We found an inconsistency in the estimated cutoffs when trying to discriminate between the different stages of AKI, with a value of 445 ng/ml for stage 1 vs. 2+3 and 177 ng/ml for stage 1+2 vs. 3. Expectations would have been for a higher cutoff value when allocating to a more severe AKI stage. This inconsistency is likely caused by the more similar distribution of NGAL values for stage 2 and 3 compared to stage 1, making separation of 1 and 2+3 easier than separation of 2 and 3 in the 1+2 vs 3 evaluation, resulting in poor specificity for the 1 vs. 2+3 evaluation. The cutoff is therefore mainly determined by the sensitivity, which is maximized at the lowest NGAL value for the stage 3 group, which also explains the very low PPV. When comparing our study`s finding to a recent meta-analysis study [43], were they assessed the predictive accuracy of pNGAL levels for AKI across 36 observational studies, it can be suggested that pNGAL cutoffs need adjustment based on the desired diagnostic purpose. Among others, they investigated severe AKI, and found a cutoff using the Youden index of 231 ng/mL with 67% sensitivity (95% CI, 46%–77%) and 89% specificity (95% CI, 76%–92%). This being much lower than the 445 ng/ml found in this current study, however with similar sensitivity and specificity. The difference in the cutoff may be explained by the meta-analysis including several criteria for definition of severe AKI in addition to the KDIGO guidelines used in the current study. Both studies do though, suggest that NGAL cutoffs are most useful in excluding severe AKI due to their high NPV. In clinical practice, these findings suggest that NGAL cutoffs should be tailored to diagnostic goals, whether to rule out or confirm severe AKI.

NGAL is an acute phase protein whose expression and release are regulated by inflammatory cytokines released as part of the inflammatory process or infection (bacterial, viral and fungal). Therefore, the levels of NGAL in plasma, increase in response to infections and inflammatory states beyond kidney-specific conditions and this should be taken in consideration when evaluating pNGAL results in patients [15]. A study investigating the link between pulmonary embolism (PE) and pNGAL levels found significantly higher pNGAL in PE patients compared to healthy controls. The authors suggested that PE may be considered an inflammatory condition, as it appears to activate various cytokines and growth factors, contributing to the elevated pNGAL levels observed in these patients [44]. While elevated NGAL levels can be part of an inflammatory response, normal levels of pNGAL indicate an absence of major systemic inflammation or significant kidney injury [45]. This highlights pNGAL’s potential in triaging ED patients, although it should be part of a comprehensive diagnostic approach. A study with 131 ST-elevation myocardial infarction (STEMI) patients found that elevated pNGAL levels measured either before or after primary coronary intervention (PCI) were independently associated with AKI [46]. This supporting the possible utility of pNGAL for future AKI in high-risk patients.

Our study suggests that a single pNGAL measurement can effectively rule out AKI at admission, providing a significant advantage in early detection and intervention in an ED setting.

A limitation is that this study is a single center study. Another potential limitation and source of selection bias is the exclusion of additional pCr measurement to determine AKI at admission. It is expected that patients with multiple pCr measurement are more likely of having an increased disease burden than does those with a single measure, as their conditions may have prompted the additional measures. A further selection was made from the lack of plasma available in the samples, however this selection is likely less bias as the amount of plasma left in each sample is more random. Additionally comparing the NGAL and AKI cohorts did not show major differences between the cohorts. Furthermore, we were unable to compare pNGAL values with the referral- nor the discharge diagnoses due to lack of information. This would have allowed to examine to what extent infection and other conditions with inflammatory response affects pNGAL levels, which could have made it possible to identify confounding factors [47–49].

## Conclusion

In summary, our investigation found that a single measurement of pNGAL has significant diagnostic utility in excluding AKI. The high NPV upon admission to the ED underscores its potential and valuable role as a rule-out marker. This allows for the stratification of ED-admitted patients by identifying those at low risk of AKI, enabling focused attention on individuals with a considerable risk of developing kidney insufficiency. Early intervention and kidney-preserving adjustments become feasible in this subgroup, potentially contributing to a reduction in CKD prevalence. Furthermore, our study suggests that utilizing mb-pCr may enhance diagnostic accuracy when pCr is used for AKI assessment.

## Supporting information

S1 Table Demographics and comorbidities in acute kidney injury- diagnostic criteria (AKI-DC) I-IV in the NGAL cohort. Abbreviation: LOS = length of stay; IHD = ischemic heart disease; CHF = cardiac heart failure; COPD = chronic obstructive pulmonary disease.
(PDF)

S2 Table pNGAL cutoff value and performance at different acute kidney injury (AKI) stages. Abbreviation: AUC = area under the curve; PPV = positive predictive value; NPV = negative predictive value. Receiver Operating Characteristic (ROC) analyses were conducted to calculate cutoff values for AKI staging based on mean baseline plasma creatinine (mb-pCr) between AKI stage 1 versus AKI stage 2 and 3 and between AKI stage 1 and 2 versus AKI stage 3. The AUC results showed that NGALs ability to discriminate AKI between stages were poor and with overlapping cutoff values between stages.
(PDF)

S3 Table NGAL cutoff values for acute kidney injury (AKI) prediction in different studies. (PDF)

S1 Fig Boxplots of mean NGAL for the different acute kidney injury (AKI) stages (1–3) based on baseline plasma creatinine (b-pCr) and mean baseline plasma creatinine (mb-pCr). The mean NGAL value for AKI staging based on b-pCr gave an impression of similar mean value for alle three stages. For AKI based on mb-pCr the mean at stage one was lower than the mean at stage two and three.
(PDF)

S2 Fig Receiver Operating Characteristic (ROC) curves over NGALs ability to stage acute kidney injury (AKI) based on mean baseline plasma creatinine (mb-pCr). Cumulative Receiver Operating Characteristic (ROC) analyses were conducted to calculate cutoff values for AKI staging based on mean baseline plasma creatinine (mb-pCr) between AKI stage 1 versus AKI stage 2 and 3 and between AKI stage 1 and 2 versus AKI stage 3. The ROC curves demonstrate that NGALs ability to discriminate AKI staging was poor (area under the curves (AUCs) ≤ 0.72).
(PDF)

## Abbreviations

AKI: acute kidney injury
AKIN: acute kidney injury network
AUC: area under the curve
b-pCr: baseline plasma creatinine
CHF: cardiac heart failure
CKD: chronic kidney disease
COPD: chronic obstructive pulmonary disease
DC: Diagnostic criteria
ED: emergency department
GFR: glomerular filtration rate
IHD: ischemic heart disease
IQR: interquartile range
KDIGO: Kidney Disease: Improving Global Outcomes
LOS: length of stay
mb-pCr: mean baseline plasma creatinine
NGAL: neutrophil gelatinase-associated lipocalin
NPV: negative predictive value
PCI: primary coronary intervention
pCr: plasma creatinine
PE: pulmonary embolism
pNGAL: plasma neutrophil gelatinase-associated lipocalin
PPV: positive predictive value
proADM: proadrenomedullin
RIFLE: Risk/Injury/Failure/Loss/End-stage
ROC: Receiver Operating Characteristic
STEMI: ST-elevation myocardial infarction
uNGAL: urine neutrophil gelatinase-associated lipocalin
