# Supplementary material for: Plasma neutrophil gelatinase-associated lipocalin as a single test rule out biomarker for acute kidney injury: A cross-sectional study in patients admitted to the emergency department
Source: PLoS One. 2025 Jan 10;20(1):e0316897. doi: 10.1371/journal.pone.0316897 (PMC11723545; doi:10.1371/journal.pone.0316897)
Supplement: S1 Table — Abbreviation: LOS = length of stay; IHD = ischemic heart disease; CHF = cardiac heart failure; COPD = chronic obstructive pulmonary disease. (PDF) [file pone.0316897.s001.pdf]

Supplementary table 1.

	AKI-DC I	AKI-DC II	AKI-DC III	AKI-DC IV
**N**	791	523	2243	2243
**Age in years (IQR)**	67 (50: 78)	67 (51: 77)	67 (51: 78)	67 (51: 78)
**LOS, median days (IQR)**	3 (1: 6)	2 (0: 6)	1 (0: 5)	1 (0: 5)
**Gender male, n (%)**	419 (53%)	283 (54.1%)	1109 (49.4%)	1109 (49.4%)
**Gender female, n (%)**	372 (47%)	240 (45.9%)	1134 (50.6%)	1134 (50.6%)
**IHD, n (%)**	96 (12.1%)	61 (11.7%)	279 (12.4%)	279 (12.4%)
**CHF, n (%)**	77 (9.7%)	43 (8.2%)	174 (7.8%)	174 (7.8%)
**Hypertension, n (%)**	195 (24.7%)	127 (24.3%)	580 (25.9%)	580 (25.9%)
**Diabetes, n (%)**	129 (16.3%)	76 (14.5%)	308 (13.7%)	308 (13.7%)
**COPD, n (%)**	127 (16.1%)	65 (12.4%)	228 (10.2%)	228 (10.2%)
**Kidney disease, n (%)**	52 (6.6%)	34 (6.5%)	111 (4.9%)	111 (4.9%)
**Liver disease, n (%)**	23 (2.9%)	18 (3.4%)	47 (2.1%)	47 (2.1%)
**Rheumatic disease, n (%)**	29 (3.7%)	17 (3.3%)	71 (3.2%)	71 (3.2%)
**Cancer, n (%)**	149 (18.8%)	123 (23.5%)	418 (18.6%)	418 (18.6%)
**Living alone, n (%)**	180 (22.8%)	115 (22%)	512 (22.8%)	512 (22.8%)
**Domestic help, n (%)**	100 (12.6%)	50 (9.6%)	255 (11.4%)	255 (11.4%)
**Nursing home, n (%)**	68 (8.6%)	47 (9%)	168 (7.5%)	168 (7.5%)
