# Supplementary material for: Plasma neutrophil gelatinase-associated lipocalin as a single test rule out biomarker for acute kidney injury: A cross-sectional study in patients admitted to the emergency department
Source: PLoS One. 2025 Jan 10;20(1):e0316897. doi: 10.1371/journal.pone.0316897 (PMC11723545; doi:10.1371/journal.pone.0316897)
Supplement: S2 Table — Abbreviation: AUC = area under the curve; PPV = positive predictive value; NPV = negative predictive value. Receiver Operating Characteristic (ROC) analyses were conducted to calculate cutoff values for AKI staging based on mean baseline plasma creatinine (mb-pCr) between AKI stage 1 versus AKI stage 2 and 3 and between AKI stage 1 and 2 versus AKI stage 3. The AUC results showed that NGALs ability to discriminate AKI between stages were poor and with overlapping cutoff values between stages. (PDF) [file pone.0316897.s002.pdf]

Supplementary table 2.

	AKI Stage	Optimal threshold	NGAL Optimal cut-off (ng/mL)	AUC (%)	Sensitivity	Specificity	PPV	NPV
NGAL	1 vs. (2,3)	0.272	445	70 [61, 78]	0.51	0.81	0.41	0.87
	(1,2) vs. 3	0.187	177	71 [60, 83]	1.00	0.43	0.11	1.00
