# Supplementary material for: Plasma neutrophil gelatinase-associated lipocalin as a single test rule out biomarker for acute kidney injury: A cross-sectional study in patients admitted to the emergency department
Source: PLoS One. 2025 Jan 10;20(1):e0316897. doi: 10.1371/journal.pone.0316897 (PMC11723545; doi:10.1371/journal.pone.0316897)
Supplement: S3 Table — (PDF) [file pone.0316897.s003.pdf]

Supplementary table 3.

**Reference**	**NGAL cutoff (ng/mL)**	**Patient group (number of patients)**	**Study type**
Di Somma et al.[12]	≥ 150	Emergency department (n=665)	Prospective cohort
Soto et al.[30]	> 97	Emergency department (n=616)	Prospective cohort
Lee et al.[35]	≥177	Cardiac arrest survivors in therapeutic hypothermia (n=279)	Retrospective observational
Haase-Fielitz et al.[36]	>150	Cardiopulmonary bypass surgery patients (n=100)	Prospective cohort
Present study	>133-193	Emergency department (n=2433)	Retrospective cross-sectional
