# Supplementary figures and images for: Plasma neutrophil gelatinase-associated lipocalin as a single test rule out biomarker for acute kidney injury: A cross-sectional study in patients admitted to the emergency department
Source: PLoS One. 2025 Jan 10;20(1):e0316897. doi: 10.1371/journal.pone.0316897 (PMC11723545; doi:10.1371/journal.pone.0316897)

Supplementary 4 figure

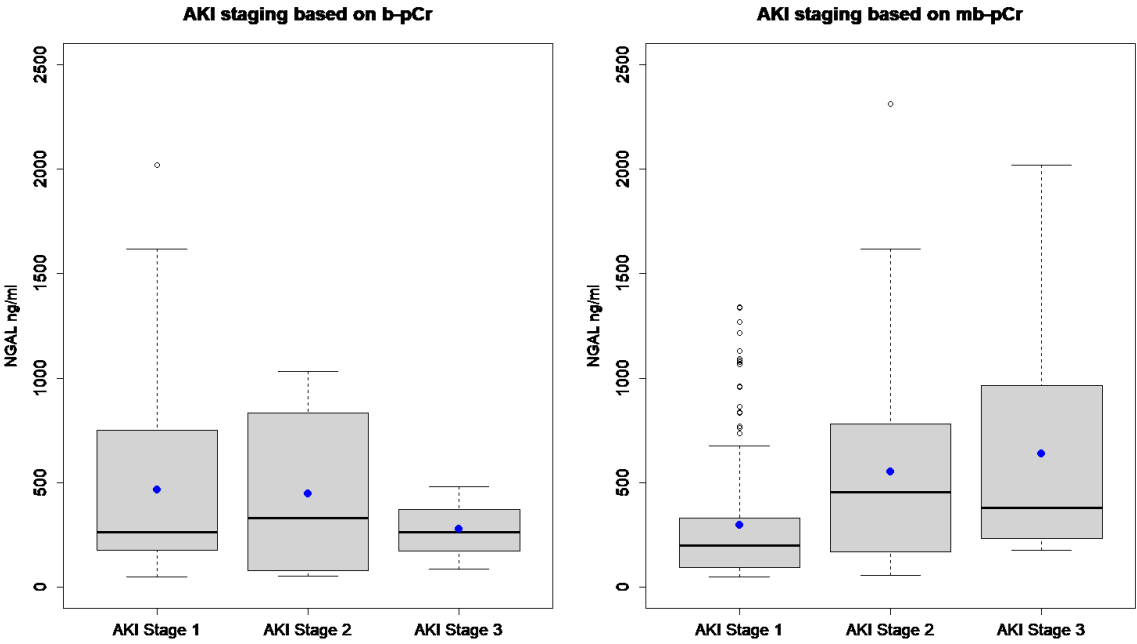

Supplement: S1 Fig — The mean NGAL value for AKI staging based on b-pCr gave an impression of similar mean value for alle three stages. For AKI based on mb-pCr the mean at stage one was lower than the mean at stage two and three. (PDF) [file pone.0316897.s004.pdf]

Supplementary 5 figure

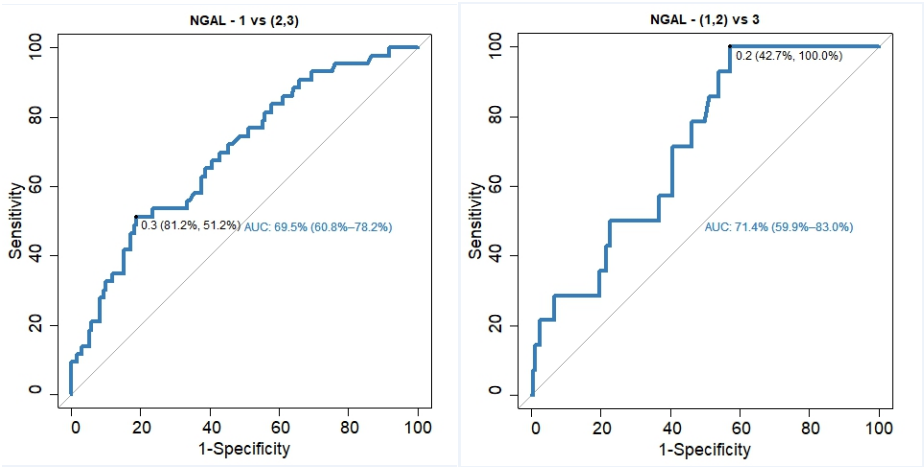

Supplement: S2 Fig — Cumulative Receiver Operating Characteristic (ROC) analyses were conducted to calculate cutoff values for AKI staging based on mean baseline plasma creatinine (mb-pCr) between AKI stage 1 versus AKI stage 2 and 3 and between AKI stage 1 and 2 versus AKI stage 3. The ROC curves demonstrate that NGALs ability to discriminate AKI staging was poor (area under the curves (AUCs) ≤ 0.72). (PDF) [file pone.0316897.s005.pdf]
